# Inservice trainings for Shiraz University of Medical Sciences employees: Effectiveness assessment by using the CIPP model

**Published:** 2015-04

**Authors:** MARYAM MOKHTARZADEGAN, MITRA AMINI, FARNAZ TAKMIL, MOHAMMAD ADAMIAT, POONEH SARVERAVAN

**Affiliations:** 1 Shiraz University of Medical Sciences, Shiraz, Iran;; 2 Education Development Center, Quality Improvement in Clinical Education Research Center, Shiraz University of Medical Sciences, Shiraz, Iran;

**Keywords:** Inservice training, Training, Effectiveness

## Abstract

**Introduction:**

Nowadays, the employees` in-service training has become one of the core components in survival and success of any organization. Unfortunately, despite the importance of training evaluation, a small portion of resources are allocated to this matter. Among many evaluation models, the CIPP model or Context, Input, Process, Product model is a very useful approach to educational evaluation. So far, the evaluation of the training courses mostly provided information for learners but this investigation aims at evaluating the effectiveness of the experts’ training programs in SUMS and identifying its pros and cons based on the 4 stages of the CIPP model.

**Method:**

In this descriptive analytical study, done in 2013, 250 employees of SUMS participated in in-service training courses were randomly selected. The evaluated variables were designed using CIPP model and a researcher-made questionnaire was used for data collection; the questionnaire was validated using expert opinion and its reliability was confirmed by Cronbach’s alpha (0.89). Quantitative data were analyzed using SPSS 14 and statistical tests was done as needed.

**Results:**

In the context phase, the mean score was highest in solving work problems (4.07±0.88) and lowest in focusing on learners’ learning style training courses (2.68±0.91). There is a statistically significant difference between the employees` education level and the product phase evaluation (p<0.001).  The necessary effectiveness was not statistically significant in context and input level (p>0.001), in contrast with the process and product phase which showed a significant deference (p<0.001).

**Conclusion:**

Considering our results, although the in-service trainings given to sums employees has been effective in many ways, it has some weaknesses as well. Therefore improving these weaknesses and reinforcing strong points within the identified fields in this study should be taken into account by decision makers and administrators.

## Introduction

Nowadays, the employees ` in-service training has become one of the core component in survival and success of any organization .In the past decade, the rapid evolution of science and information technology, not only has heighten the need for but also mandate such trainings. Living in the age of information and technology advancement, the employees can no longer fulfill their diverse duties by relying on their old and out dated knowledge and skills. Moreover, the organizations also cannot remain indifferent to these advances, since they must compete with parallel organizations which utilize these new technologies. These facts has pointed out a very important need for organizational survival and improvement which is essential designing, implementing and constant evaluating of in-service educations. In addition, by extending occupational knowledge, attitude, and teaching group work through cooperative learning, the in-service training can enhance human resources capability and help them to do their job more efficiently (1). It also will result in lifelong learning since philosophically speaking, in - service education has origins in it (2).

Unfortunately, despite the importance of training evaluation, a small portion of considerable resources are actually allocated to this matter. What’s more, many expensive in- service training programs which are even designed thoughtfully lack evaluation (3).

Homen believed that whatever is done to gather information, improve training problems and provide feedbacks for managers can be considered as training evaluation. Therefore, in order to make intelligent decisions and as a result improve the quality of training programs an evaluation based on these factors must be done. Such a feedback, if designed properly, will guarantee the success of training programs (4).

As Stuffelbeam has proven in 1983, among many evaluation models, the CIPP model or Context, Input, Process, Product model is a very useful approach to educational evaluation which as a standard, provides a systematic and constructional way of evaluation in many training programs. Based on CIPP model the most important goal of evaluation is the training courses improvement rather than its confirmation. .In addition, instead of focusing only on an individual improvement, this model offers information which can be used by decision makers in a training institute for ongoing evaluation of the programs. By providing an organized feedback of the current affairs, this model helps the managers to prioritize the essential needs and allocate available resources to more effectual activities (5, 6).

In context evaluation, primary programming and the program environment are examined. Since in this phase, the program may have not been started yet, the need to implement the program  and required facilities may also be examined (7).

The input evaluation phase which starts after the execution approval includes: program time table, facilities and human resources management (7). The goal of input evaluation is facilitating the implementation of the program designed in the context phase. In addition this phase also focuses on human and financial facilities, politics, training changes, instructional strategies, obstacles and limitations of the training system (8).

In the process evaluation phase, the program itself goes under close observation and the changes of input during the program and the later on output is investigated. In fact, it is a quality control phase during its execution (7). The performance of process evaluation is largely depended on the implementation of the two previously mentioned phases; in other words, if the context and input evaluations are  done properly, the implementation of  the program  is defiantly easier and more successful; as a result the process  phase face less obstacles (8).

The final phase or product evaluation is considered as the goal of evaluation. By demonstrating all the desired and undesired outcomes, this phase shows what has or has not been done (7). As Stuffelbeam put it, product evaluation is measuring and judging the achievement of predefined goals. In product evaluation this measurement and interpretation is done not only at the end of the program but also during the program execution and completing each task. This is due to the periodic feature of   the evaluation process, in other words evaluation is a process that should be done constantly and the results of each phase should be utilized to improve the previous phase and program the next phase. In some cases product evaluation is expanded until it covers long term effects of a program and its positive and negative products (8).

So far, the evaluation of the training courses mostly provided information for learners but this investigation aims at evaluating the effectiveness of the experts’ training programs in Shiraz University of Medical Sciences based on the CIPP model, and identifying weak and strong points of the program, and providing results for administrators and decision makers, and can obtain guidelines for designing better quality programs. 

## Methods

In 2013, this analytical and illustrative study was done on all the employees of Shiraz University of Medical Sciences (2219) who took part in different in-service trainings. We used Kerjesy and Morgan formula for calculating the sample size.

In an attempt to increase the accuracy of the study and compensate for possible low response rate, 10 percent was added to the actual sample size. After gaining informed consent , 250 employees which were selected randomly entered the survey .A  questionnaire based on Stuffelbeam’ s CIPP evaluation model was designed (9). This questionnaire consists of  two parts: The first part collect the  demographic data such as :gender, education level, age, employment type, work experience, field of study, type of job and place of work ;whereas the second part which contained 62 ( 58 quantitative and 4 qualitative ) questions gathered the effectiveness data . The employees ranked each quantitative item based on a five rating scale which was :( 1= very low, 2= low, 3= average, 4= high, 5= very high). Achieving 3 points was considered the cut point of effectiveness for each phase which meant that the effectiveness is undesirable if the average of points given to the items in each phase is less than 3 (1-2.99) and desirable if it is 3 or more (3-5) ,providing that the (p<0.001) in each case.

The qualitative questions were open –ended and used for gathering personal opinions and suggestions. The questionnaire`s questions were based on four phases of CIPP model and previous studies,. These phases are:

1. Context Phase: Training infrastructure, opportunities, satisfaction, training needs assessment, training resources, learning styles, location status, and manager support.
2. Input Phase: Staff, training content, program designing, budget and financial resources, training environment, supports staff, and instructors.
3. Process: Training process, learning, number of instructors, instructors’ performance, support and encouragement, performance of the library and announcement.
4. Product or outcomes Phase: The quality of educational content, the rate of reaching goals, effectiveness of trainings, enhancing the knowledge and skills, the motivation for getting educational certificate, evaluation of trainees' information and empowering the staff.

Questions 1 to 10 evaluate the context component, Questions 11 to 25 evaluate the input component, Questions 26 to 48 evaluates the process component, Questions 49 to 58 evaluates the product component, and Questions 59,60,61,62 measures the results, qualitatively. The reliability of the questionnaire was determined by experts through Cronbach’s alpha test. The cronbach’s alpha coefficient was 0.89 which suggests that the questionnaire has the required reliability. The validity was also established using experts opinion. The questionnaires were distributed and collected via face to face or email approach. The data were then analyzed using descriptive and inferential statistics including distribution of population, mean, standard deviation, correlation coefficient, and t-test. 

## Results

In the present study, 68% of the participants were women and 32% were men. 163 participants (67.2%) had Bachelor’s, 57 (23.4%) had associate and 23(9.4%) had Master’s and PhD degrees. The mean and standard deviation of the study participants’ age and work experience were (36.3±6.14) and (11.25±6.79), respectively. The employees enrolled in the study were from deferent departments such as healthcare, administrative - financial, educational- cultural and technical-engineering departments. The gender affected the process phase `s results significantly (p=0.045); in contrast with the other phases in which the gender didn’t play any role (p>0.001). In the product phase , the educational degree of the employees which included Associate , Bachelor’s , Master’s and PhD degrees   significantly affected the results (p<0.001).

Quantitative data showed that in the context phase, the mean score was highest in solving work problems (4.07±0.88) and lowest in focusing on learners’ learning style training courses (2.6 ± 0.94). Although the total score achieved in the context phase was 3.17 overall, this phase didn’t have the necessary effectiveness (p>0.001) (Table 1).

**Table 1:** The context phase

Evaluation component	Research question: How are the staff training programs with respect to evaluation of context?		
**Context**	**Variable**	**Num**	**Mean±SD**
	Training as solving work problems	246	4.07±0.88
	Interested in participating in training courses	250	4.02±0.95
	The status of training environment	249	3.39±0.87
	Appropriate opportunity for using skills	248	3.12±0.86
	Facilities and substructures of electronic education	248	2.69±0.94
	Focusing on learners’ learning style	246	2.68±0.91
	Support and encouragement from managers	247	2.89±0.95
	Availability of educational resources	247	2.87±0.99
	Appropriate environment from managers and executive administrators in order to use educational content	249	2.87±0.95

Mean from 5

In the input phase, the maximum mean score was related to holding courses in office hours and the instructors’ interest in teaching (3.6±0.731) and the minimum mean score was related to needs assessment of training courses (2.4±1.63). The total score was reported to be 3.03 Overall, but like the context phase, this phase also lacked the necessary effectiveness (p>0.001) (Table 2).

**Table 2:** Frequency distribution, mean score and response rates of questions about input

Evaluation component	Research question: How are the staff training programs with respect to evaluation of input?		
Input	**Variable**	**Num**	**Mean±SD **
	Holding courses in office hours	249	3.63±1.14
	Instructors’ interest in teaching	247	3.62±0.731
	Using competent instructors	249	3.26±0.871
	Training environment( hotness, coldness, …)	250	3.26±0.929
	Using competent training administrators and liaisons	249	3.46±0.884
	Conformity of facilities with educational needs	250	3.10±0.789
	Panning and programming training programs before holding courses	243	3.19±0.819
	Scientific capability of Instructors in teaching	247	3.25±0.812
	Scientific capability of library administrators	228	3.01±0.890
	Compiling training contents based on the needs of the organization and learners	250	2.69±0.924
	Allocation of budget and financial resources	226	2.65±0.835
	Allocation of training resources ( books, …)	244	2.69±0.897
	Needs assessment of training courses	246	2.47±1.063
	Setting programs according to work activities of learners	246	2.84±0.988
	Sufficient resources and facilities in libraries	237	2.38±0.929

Mean from 5

In the process phase of the evaluation, using new educational methods and technologies had the maximum (3.50±.862) and sufficient budget and facilities for educational needs had the minimum mean score (2.6±0.855). The total score was reported to be 3.15. Overall and it was statistically significant (p<0.001), therefore it can be concluded that this phase had the necessary effectiveness (Table 3).

**Table 3:** The process phase: Frequency distribution, mean score and the questions ` response rate

Evaluation component	Research question: How are the staff training programs with respect to evaluation of process?		
**process**	**Variable**	**Num**	**Mean±SD**
	Applying new educational technologies( slides, overheads, …)	248	3.52±0.862
	Announcing the dates of holding training courses	245	3.43±0.962
	Conformity of number of instructors with teaching the educational content	243	3.40±0.799
	Introducing the goals of training programs	249	3.07±0.855
	Focusing on effective teaching methods	246	3.01±0.815
	Quality of educational tools	248	3.15±0.854
	Motivating learners to learn	247	3.03±0.896
	Using practical examples during teaching	246	3.09±0.898
	Up to date content of courses	245	3.31±0.860
	Conformity of workshop duration with training subjects	242	3.04±0.910
	Observing a logical continuation between subjects	244	3.17±0.844
	Allowing learners to express opinions	247	3.36±0.978
	Encouraging learners to learn	245	3.33±0.900
	Providing support services	245	3.24±0.865
	Meeting the needs and solving educational problems of learners	249	3.22±0.875
	Work and technical conformity of learners with each other	248	3.14±0.925
	Conformity of educational environment with the number of learners	247	3.29±0.904
	Using problem solving teaching method	243	3.00±0.876
	Focusing on learner based teaching method	243	3.25±0.876
	Focusing on skills and practical components of training	244	3.06±0.906
	Conformity of training duration with subjects	244	2.93±0.889
	Conformity of education content with the allocated time	244	2.87±0.882
	Sufficient budget and facilities for educational needs	239	2.67±0.855

Mean from 5

In the product phase, “the quality of materials offered” had the highest mean score (3.2±0.749) while the lowest mean score belonged to“the offering feedback item” (2.8±0.966). The total score was reported to be 3.13, Overall, and since it was statistically significant, the product phase has the necessary effectiveness (P<0.001) (Table 4).

**Table 4:** The product phase: Frequency distribution, mean score and the questions` response rates

Evaluation component	Research question: How are staff training programs with respect to evaluation of product?		
**Product**	**Variable**	**Num**	**Mean±SD**
	Receiving certificate of education	246	2.55±1.011
	Quality of materials offered	248	3.27±0.749
	Achieving goals of the program	244	3.05±0.796
	Effectiveness of education in action	246	3.06±0.904
	Desirable increase of knowledge	246	3.26±0.907
	Meeting scientific needs	243	3.04±0.933
	Evaluating information by performing pre test and post test	243	3.13±1.027
	Work and occupational empowerment	247	3.18±0.994
	Offering feedback	243	2.83±0.966
	Using the benefits of training programs by managers and executive administrators	241	2.97±1.014

Mean from 5

The CIPP Model full reports based on the participants answer are as follows:

 Regarding the question “how to hold a better training course”, the most effective factors included:

Appropriate place selection   with respect to proximity to workplace, holding virtual and distance courses, motivating learners and providing appropriate situation for attending training courses, in the context phase. Moreover,  utilizing  instructors from inside and outside of the organization, needs assessment, holding courses based on the employees work requirements  and degrees, programming before implementation, holding the courses in  office hours, Allocating  enough budget, educational resources and facilities and general environment of the courses were the most important factors mentioned  in the  input phase, and last but not least  regarding the process phase, the main contributing factors were : holding practical courses, applying new educational methods such as  learner- based teaching, reducing the number of learners, allocating enough time to each course and announcing the dates  before head .

In response to the question” whether evaluation can result in the improvement of programs and why” in the product phase:

The most influential factors in in-service training evaluation included:

1. Methodical evaluation
2. Weaknesses and strong points identification
3. Attending these training programs should have a better motivation rather than just receiving a certificate in education
4. Improving the quality of education and level of knowledge and science
5. Improving programs
6. Offering feedbacks

## Discussion

The results of the current study have shown that the effectiveness of in-service training depends on various factors. Therefore, in order to be effective, these factors must improve. Regarding the training courses presented to SUMS` employees, the CIPP evaluation done in this survey indicated that, there are some strong points and some weaknesses. 

According to our study, high interest in taking these courses and the training which result in solving work problems are the program strong points while the weaknesses included : the lack of educational  facilities for holding virtual training courses, inaccessibility of educational resources, inappropriate environment for applying educational materials, and lack of support and encouragement by administration .These results confirm the  results of other studies which aimed at investigating the weaknesses of in-service training courses such as Zandvarian et al.’s (2009), Naeeni et al.’s (2006) and Kazemi et al.’s (2009) studies (10-12) .

Like Hosseini et al in 2008 ,our study have found that  factors such as allocating budget, financial and educational resources, needs assessment of training courses and take the staffs` problems in to consideration while choosing the subject and content of these courses, to be  the most important issues in the component of input (1).

The results of the present study showed that the most influential factors which decrease the effectiveness of the programs are ignoring needs assessment procedures, educational-welfare conditions and facilities (12). Also, our results are in agreement with Mahmoodi et al (2012) and Yarmohammadian et al.’s. (2011) studies in which needs assessment are mentioned as the greatest change of innovation in sustaining the  in-service training programs which is lower than expected (2, 13). Decision makers and education programmers should pay more attention to the necessity of needs assessment that is the first and most important stage in each organization (13).

One of other effective factors is to utilize instructors from inside and outside of the organization. In this way, the program can benefit from the insiders awareness due to their familiarity with the organization problems and also the specialty and experience of outsiders in special fields. Other factors include: programming before implementation and holding courses compatible with the employees’ work and degree during their work hours which are in agreement with Mahmoodi et al.’s study in 2010 (13).

Other training effectiveness obstacles included: lack of budget and facilities for educational needs, insufficient educational contents and time allocated to them. Items such as goals introduction methods, effective teaching style, quality of educational tools, learners’ motivation enhancement and the use of practical examples during teaching and relatively up-to-date contents must also be taken in to consideration and revise. Khotami’s study (2012) who also conducted an evaluation based on CIPP model in Saudi Arabia also emphasized on the importance of facilities and equipment, training environment and capability of instructors as a useful controlling model (14).

Other effective factors of this component may include holding practical courses, applying new teaching methods, reducing the number of learners in training sessions, learner-based training, allocating enough time for each course and announcing the time of holding courses that are in agreement with a study done by Tazakori et al.’s (2010) which reported that new methods of teaching were not used (15).

In the product phase, the employees believed that one of the main weaknesses of the evaluation is the fact that many attend these courses with sole purpose of receiving educational certificate. Other weak points in this phase included the lack of utilization of program results by administrators and their ignorance of such programs’ benefits, as well as lack of feedbacks to staffs. Likewise, in Hosseinpour et al’s study (2012) which also found motivations such as a guaranteed certificate for work promotion and raise upon fulfillment of the course as one of the trainings weak points, since it could consequently lead to lack of active participation in training and less effective program (16).

Also, results from Akhlaghi and Yarmahmmodian (2011) and Heidari et al.’s. (2005) studies showed that evaluation of the product phase was not desirable and even lower than expected indicating some problems; therefore a sustainable evaluation and identification of weak and strong points of programs should be used to improve the quality level of training courses (17, 18).

## Conclusion

In conclusion, although the in-service trainings given to sums employees have been effective in many ways, they also have some weaknesses especially in the input and context phases, in which more effectiveness was expected. The product and process phases of the training, however, were desirably effective.Therefore, Improving these weaknesses and reinforcing strong points within the identified fields in this study should be taken into account by decision makers and administrators. In this regard it seems that to enhance the positive points of these training courses, an environment of creativity and productivity in all phases must be created and actively supported by the administration and those in charge of in-service training program .If done correctly and continuously, this may also lead to weak points` improvement. Moreover, needs assessment, electronic learning facilitation and the employees points of view regarding the time and place and content of the trainings must be taken under consideration in 
